# Supplementary material for: Splice-Junction-Based Mapping of Alternative Isoforms in the Human Proteome
Source: Cell Rep. Author manuscript; Available in PMC 2020 Jan 15. (PMC6961840; doi:10.1016/j.celrep.2019.11.026)
Supplement: 2 [file NIHMS1546469-supplement-2.zip › DF1/PXD010154/Lung/Lung_28_RACK1_ANCKLTLKGHNGWVTQIATTPQFPDMILSASR.pdf]

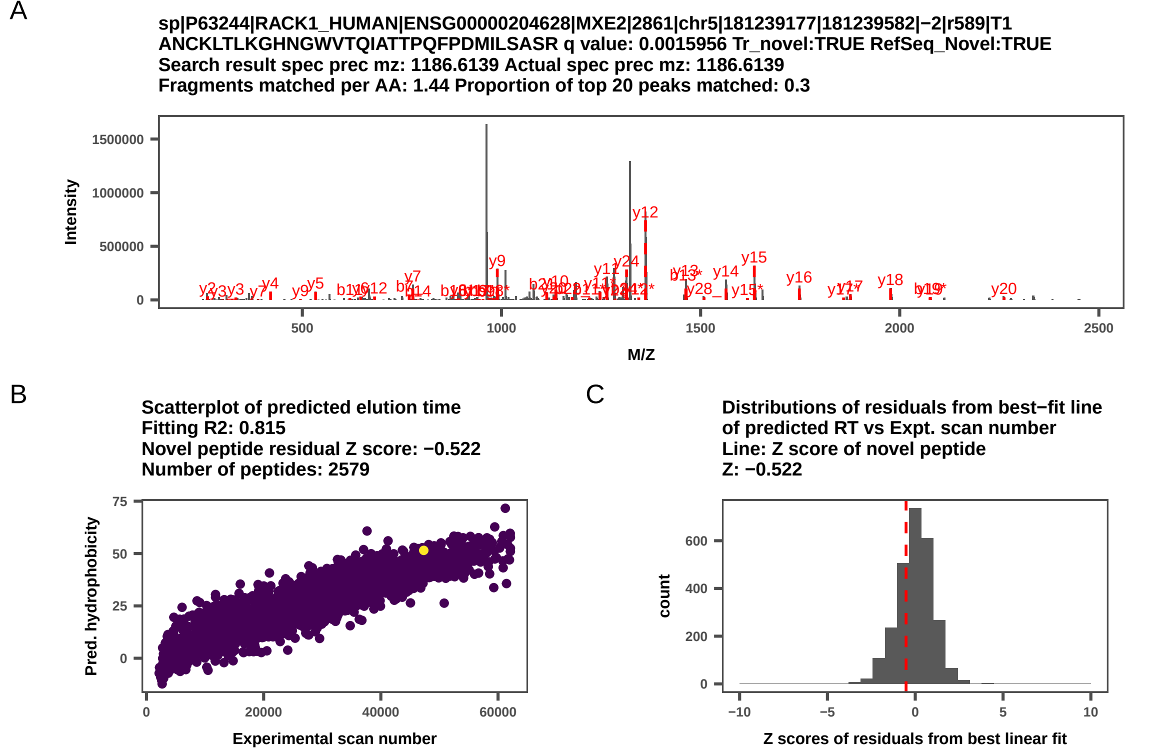
A
sp|P63244|RACK1_HUMAN|ENSG00000204628|MXE2|2861|chr5|181239177|181239582|−2|r589|T1
ANCKLTLKGHNGWVTQIATTPQFPDMILSASR q value: 0.0015956 Tr_novel:TRUE RefSeq_Novel:TRUE
Search result spec prec mz: 1186.6139 Actual spec prec mz: 1186.6139
Fragments matched per AA: 1.44 Proportion of top 20 peaks matched: 0.3
Intensity
1500000
1000000
500000
0
500
1000
1500
2000
2500
M/Z
y12
y9
y15
y14
y16
y18
y20
B
Scatterplot of predicted elution time
Fitting R2: 0.815
Novel peptide residual Z score: −0.522
Number of peptides: 2579
Pred. hydrophobicity
75
50
25
0
0
20000
40000
60000
Experimental scan number
C
Distributions of residuals from best−fit line
of predicted RT vs Expt. scan number
Line: Z score of novel peptide
Z: −0.522
count
600
400
200
0
−10
−5
0
5
10
Z scores of residuals from best linear fit
